# Comment on: Molecular Functions of Thyroid Hormone Signaling in Regulation of Cancer Progression and Anti-Apoptosis

**DOI:** 10.3390/ijms21082684

**Published:** 2020-04-13

**Authors:** Magdalena Szaryńska

**Affiliations:** Department of Histology, Medical University of Gdansk, Debinki 1, 80-210 Gdansk, Poland; mszarynska@gumed.edu.pl; Tel.: +48-58-349-14-37; Fax: +48-58-349-14-19

**Keywords:** thyroid hormones, thyroid hormone receptors, T3, cancer

Dear Editor,

I have found the article by Liu YC et al. very interesting, with great cognitive value [1]. The manuscript extensively reviewed complex aspects regarding thyroid hormones activity in both normal and cancer cells, with a focus on the functions of thyroid hormone receptors. It presents a detailed description of hypothalamic-pituitary-thyroid axis, with specific proteins engaged in the regulatory mechanisms. Hypothyroidism seems to exert a protective role in some tumor types, however hyperthyroidism can increase the risk of cancer transformation and promote the development of unfavorable features of cancer cells. However, the relationship between thyroid gland condition, thyroid hormones activity and cancerogenesis remains controversial and requires more efforts, because observations concerning different groups of patients are not consistent. Since the existence of cancer stem cells (CSCs) was proven for most of the tumor types, the analysis of relations between endocrine system and steps of cancer transformation seems to be crucial for potential therapeutic implications. The major roles of CSCs focus on driving the incidence, mortality and metastasis of tumors [2].

I found the manuscript by Liu YC et al. very helpful in my search for information concerning these issues, however, I found a confusing aspect in Figure 1. The table summarizing the features of four thyroid hormone receptors (TRs) reports that TRβ2 has no T3 binding abilities, although it is commonly known that a lack of thyroid hormone (especially T3) binding domain is a characteristic of TRα2 [3,4].

T3 action is completely dependent upon its receptors [5,6], which belong to the nuclear receptor superfamily known as ligand-activated transcription factors. These receptors are formed from a single polypeptide chain that is folded into three functional domains: an amino-terminal domain (A–B domain), a central DNA-binding domain (DBD) and a carboxyl-terminal ligand-binding domain (LBD) [5]. The binding of T3 changes the configuration of C-terminal helix (H) 12, blocks a hydrophobic surface that binds corepressors and completes a coactivator binding surface (activation function 2, AF-2), which includes residues from H3, H5 and H12. AF-2 acts as a docking site for the short α-helical coactivators of thyroid hormone target genes transcription [7]. The LBD is responsible for the specificity of the receptor, for its ligand and its capacity to form either homodimers or heterodimers with other members of the nuclear receptor superfamily [8].

There are three main T3-binding isoforms of receptors. TRβ1 and TRβ2 are translated from alternatively spliced mRNAs generated from a single gene, whereas TRα1 is encoded by a separate gene, but shares a very high structural homology with the TRβ isoforms. TRα2 has a distinct C-terminal extension and does not bind T3 [9].

If I may offer a suggestion to Editors, it might be a good idea to correct the data in Figure 1. It would be beneficial to mark the presence or absence of AF-2 in the LBD of all mentioned TRs (in graphical form). In my opinion, this change would make the Figure much more informative, precisely showing which receptor has the ability, or not, to bind the T3 thyroid hormone. I suggest the correction of the current table, as follows:
**Thyroid Hormone Receptor Isoforms****T3 Binding Ability****Presence of AF-2**TRα1YESYES**TRα2****NO****NO**TRβ1YESYESTRβ2YESYES

I believe that my suggestion would improve the scientific quality of the Liu YC et al. manuscript and would be helpful to the readers of IJMS. However, extensive research must be conducted to explore the T3 impact on cancer cells in in vivo and in vitro protocols, to provide full knowledge, before confirming that T3 supplementation is a profitable element of the adjuvant therapy of cancer patients.
